# Development and validation of a stability indicating RP-HPLC-DAD method for the determination of bromazepam

**DOI:** 10.1371/journal.pone.0244951

**Published:** 2021-03-10

**Authors:** Hany W. Darwish, Nesma A. Ali, Ibrahim A. Naguib, Mohamed R. El Ghobashy, Abdullah M. Al-Hossaini, Maha M. Abdelrahman

**Affiliations:** 1 Department of Pharmaceutical Chemistry, College of Pharmacy, King Saud University, Riyadh, Kingdom of Saudi Arabia; 2 Faculty of Pharmacy, Analytical Chemistry Department, Cairo University, Cairo, Egypt; 3 Analytical Toxicology Laboratory, Forensic Medicine Authority, Justice Ministry, El Sayeda Zeinab, Cairo, Egypt; 4 Department of Pharmaceutical Chemistry, College of Pharmacy, Taif University, Taif, Saudi Arabia; 5 Faculty of Pharmacy, October 6 University, October 6 city, Giza, Egypt; 6 Faculty of Pharmacy, Pharmaceutical Analytical Chemistry Department, Beni-Suef University, Beni-Suef, Egypt; Bhagwan Mahvir College of Pharmacy, INDIA

## Abstract

A reliable, selective and sensitive stability-indicating RP-HPLC assay was established for the quantitation of bromazepam (BMZ) and one of the degradant and stated potential impurities; 2-(2-amino-5-bromobenzoyl) pyridine (ABP). The assay was accomplished on a C_18_ column (250 mm × 4.6 mm i.d., 5 μm particle size), and utilizing methanol-water (70: 30, v/v) as the mobile phase, at a flow rate of 1.0 ml min^-1^. HPLC detection of elute was obtained by a photodiode array detector (DAD) which was set at 230 nm. ICH guidelines were adhered for validation of proposed method regarding specificity, sensitivity, precision, linearity, accuracy, system suitability and robustness. Calibration curves of BMZ and ABP were created in the range of 1–16 μg mL^-1^ with mean recovery percentage of 100.02 ± 1.245 and 99.74 ± 1.124, and detection limit of 0.20 μg mL^-1^ and 0.24 μg mL^-1^ respectively. BMZ stability was inspected under various ICH forced degradation conditions and it was found to be easily degraded in acidic and alkaline conditions. The results revealed the suitability of the described methodology for the quantitation of the impurity (ABP) in a BMZ pure sample. The determination of BMZ in pharmaceutical dosage forms was conducted with the described method and showed mean percentage recovery of 99.39 ± 1.401 and 98.72 ± 1.795 (n = 6), respectively. When comparing the described procedure to a reference HPLC method statistically, no significant differences between the two methods in regard to both accuracy and precision were found.

## Introduction

One of the extensively used derivatives of the 1,4-benzodiazepine series is the compound bromazepam **(**7-bromo-5-(pyridyl-2-yl)-1,3-dihydro-2*H*-1,4-benzodiazepin-2-one) [1]. BMZ is reported to have similar properties to those of diazepam. The compound was used in the short-term treatment for some disorders including anxiety disorders that occur alone or associated with insomnia [2]. Benzodiazepines are a class of compounds known to have sedative, hypnotic effects. Besides, benzodiazepines show muscle relaxant, anticonvulsant and amnesic properties relatively safe when compared with other sedative drugs [3]. The inhibitory action of benzodiazepines on the CNS is a result of interaction of these compounds to the gamma-aminobutyric acid (GABA) receptors within the brain [4].

Bromazepam is among one of the widely recommended benzodiazepine derivatives for treating insomnia and anxiety [5,6]. The British Pharmacopoeial (BP) assay of BMZ in bulk powder is by anhydrous titration with perchloric acid in acetic anhydride, using potentiometry for the detection of the endpoint [7].

For the detection and determination of benzodiazepines in pharmaceutical formulations and related biological matrices, various analytical techniques have been reported [8–10]. Many publications can easily be found for BMZ analysis to study and measure therapeutic or toxic blood levels of the drug in serum [11,12]. These reported methods also reveal many analytical techniques for BMZ determination either alone or together with other benzodiazepines, in both pharmaceutical or biological matrices. These techniques include HPLC [13–24], LC-MS [25,26], GC-MS [27], TLC [28], capillary electrophoresis [29], spectrophotometry [30–32], ion-selective electrode [33–35], potentiometric [36] and voltammetric [37,38] methods.

**Fig 1:**
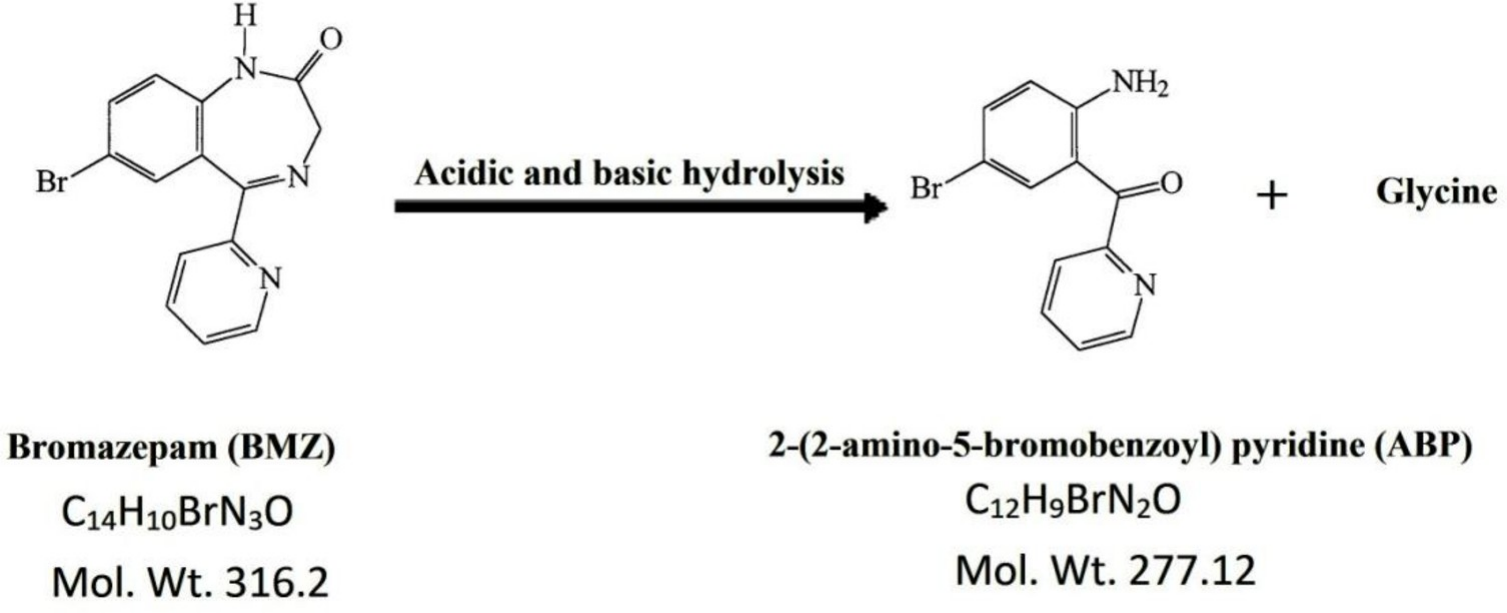
Degradation pattern of bromazepam (BMZ) by acidic and alkaline hydrolysis forming the degradate (ABP).

Similar to almost all of the benzodiazepine derivatives, BMZ can be subjected to hydrolysis in acidic aqueous solutions forming degradation products. Due to the widespread use of BMZ, a study of the kinetics and mechanism of its hydrolysis is considered a critical issue. Over the past decades, several publications showed an interest in stability studies of BMZ, especially degradation of the drug due to hydrolysis, which cause the formation of the 2-(2-amino-5-bromobenzoyl) pyridine (ABP), **Fig 1** [18,39,40]. Beside the BP [7] defines 2-(2-amino-5-bromobenzoyl) pyridine (ABP) as a possible impurity in pure bromazepam powder, the main metabolic pathway of bromazepam includes C3 hydroxylation and heterocyclic ring hydrolysis producing two metabolites 3-hydroxy bromazepam and ABP metabolites that excreted in the urine [38,41,42].

The main interest of presented paper is to evaluate BMZ stability in line with the applicable guidelines of ICH utilizing a full validated stability-indicating method after subjecting the investigated drug to a wide range of stress conditions [43,44]. Moreover, the development of a validated stability-indicating HPLC method for BMZ quantitation in raw material and tablets is the second goal. It is worth mentioning that recently, our research team published stability-indicating spectrophotometric methods for assaying BMZ in addaition to its degradant [45]. But the previous work was suitable only for resolving such a binary mixture of BMZ and its degradation product. Consequently, were directed this work for developing RP-HPLC with Photo-Diode Array detection because of its well known higher selectivity when compared to spectrophotometric methods. Additionally, The described HPLC methodology is favorably applied in quality control laboratories.

This method isn’t only for BMZ quantitation in the existence of its degradant ABP, which is considered one of its potential impurities [7] and its main benzophenone metabolite, but also for ABP determination with excellent accurateness even though the existence of great similarity in their chemical structures, **Fig 1**.

## Experimental

### Instrument

The chromatographic assay was established on an “Agilent 1260 Infinity HPLC system (Agilent Technologies, Germany)” that was fitted with an “Agilent 1260 Infinity preparative pump (G1361A)”, temperature of column was kept at 30°C by an “Agilent 1260 Infinity Thermostated column compartment (G1316A)”. Samples were introduced using an “Agilent 1260 Infinity preparative Autosampler (G2260A)”. The HPLC instrument was connected to an Agilent 1260 Infinity Diode array detector VL (G131SD). A reverse-phase “ZORBAX Hypersil BDS” C18 column 250 mm × 4.6 mm i.d. and 5 μm particle size (ThermoElectron Corporation, USA) was used. LC-solution software was utilized for recording and analyzing the chromatograms. For pH adjustment and sonication, “Jenway 3505 pH meter (Staffordshire, UK)” and “Sonix TV ss-series ultrasonicator (USA)”, were utilized respectively.

The IR spectra were determined on “Shimadzu IR 435 spectrophotometer (Shimadzu Corp., Kyoto, Japan)”. The mass determination was done on “Triple quadrupoles mass spectrometer” with API source, Agilent 6400, MassHunter software operated by Pentium 3 (40 MHz) processor (Hp, USA).

### Material and reagents

Bromazepam standard (99.91% purity) was generously provided by the “Egyptian International Pharmaceutical Industries Company (Cairo, Egypt)”. Lexotanil^®^ tablets (3 mg batch No. M1139B01) produced by “La Roche S.p.A. Milan, Italy”, and Calmepam^®^ tablets (batch No. A506716) produced by “GlaxoSmithKline SAE. (Cairo, Egypt)”, were procured from the Egyptian market. HPLC grade methanol was purchased from Sigma-Aldrich Chemical (Germany). Analytical grade chloroform, sodium hydroxide, acetone, 30% hydrogen peroxide and hydrochloric acid solutions were all purchased by “El-Nasr Pharmaceutical Chemicals Co. (Cairo, Egypt)”. Water for injection B.P. 2003 was received from Egypt “Otsuka Pharmaceutical Co. (10^th^ of Ramadan city, Egypt)”. Solvents were filtered by a “Sartorius Stedium Biotec. GmbH. membrane filters (0.45 μm) (Goettingen, Germany)”. Cellulose acetate syringe filters with 0.45 μm pore size purchased from “Gemma Medical (Barcelona, Spain)”.

#### Standard solutions

BMZ and ABP stock solutions (1 mg mL^-1^) were prepared in absolute methanol. BMZ and ABP working solutions (100 μg mL^-1^) were prepared in the proposed mobile phase (a mixture of methanol: water (70:30 v/v)).

### Degradation studies

Different forced degradation studies were performed on BMZ including acidic, alkaline and oxidative degradation studies. Literature [46] stated that BMZ is stable in light, so light-degradation studies were not performed in our work.

#### Acidic hydrolyis and preparation of degradant (ABP)

As stated previously [18], the production of the degradation product (ABP) by acid hydrolysis was achieved by dissolving 1.00 gm of pure BMZ in the least volume of methanol. The second step was the addition of 50 mL of 1N HCL that were all refluxed and protected from light for 3 h. The reaction was followed using TLC, with a developing system that contained a 4:1 chloroform-acetone solution mixture, to monitor the full disappreance of BMZ spot and emergence of a new spot of ABP. After complete degradation, the solution was neutralized by dropwise adding 10 N NaOH solution until the production of a yellow precipitate of ABP, which was kept in the refrigerator overnight. The ABP precipitate was then filtered and washed with 0.01 N HCL to dissolve any BMZ still not degraded. ABP was then washed with distilled water several times then dried in oven at 70°C. Confirmation was acquired by subjecting both BMZ and ABP to IR and Mass spectrometry.

#### Alkaline degradation

A weighted amount of pure BMZ (0.50 gm) was to be dissolved in the least volume of methanol, then refluxed away from light with 50 mL of 1N NaOH solution for 3 hours. The alkaline degradation reaction was monitored via TLC, similar to acidic degradation procedure. Once again, the same degradation product (ABP) was formed. However, to a lesser amount than what was produced by the acidic degradation method.

#### Oxidative degradation

Compare to both the acid and alkaline degradation methods above, a very limited quantity of ABP was produced by oxidative degradation of BMZ. This was achieved by putting 0.50 gm of BMZ in a 10 mL conical flask containing 30% w/v hydrogen peroxide. Then the mixture was located over a water bath (thermostatically controlled) at temperature of 80°C for 12 h. The oxidative degradation reaction was monitored by runningTLC as before.

### Procedure

In our study, chromatographic separations were effected using an isocratic mode with a C_18_ column. The mobile phase composed of (70:30 v/v) mixture of methanol (phase A) and water (phase B), at a constant flow rate of 1 mL/min. The eluate was scanned at a set wavelength of 230 nm at RT. Each injection run was replicated three times, with an injection volume of 20 μL. Total run time of each sample injection was approximately 6 min, and quantification of the components under invesigationwas achieved using the total peak areas of the investigated components.

### Linearity and calibration curves’ construction

Accurate aliquots equal to 10–160 μg of both BMZ and its degradant ABP have been transferred from their respective working standard solutions (100 μg mL^-1^) to different sets of 10 mL measuring flasks and then diluted with mobile phase to the mark. For each concentration triplicate injections were performed. The relative peak areas were determined by dividing the peak areas of each component at the corresponding peak area at 8 μg mL^-1^ as an external standard. Thereby each component’s calibration curve was constructed by using its relative peak areas, and later regression equation was created.

### Application to the pharmaceutical formulation

A fine powder has been made by first weighing and then grinding 30 Lexotanil^®^ and Calmepam^®^ tablets individually. Precisely weighed portion containing 50 mg of BMZ has been transferred into separate two volumetric flasks, each containing 30 mL of methanol. The solution mixtures were then sonicated for 30 min, cooled and then completed with methanol to a final volume of 50 mL. The prepared solutions were filtered and then further diluted with the HPLC mobile phase to reach a standard solution concentration of 100 μg mL^-1^. The detailed procedure under “linearity and calibration curve construction” was preceded and BMZ concentrations were determined by applying the regression equation, and the recovery % were computed. For implementing the standard addition step, the fine powdered Lexotanil^®^ and Calmepam^®^ tablets and pure BMZ were separately well mixed together before continuing the procedure referred to above.

## Results and discussion

### Structure elucidation of BMZ degradant (ABP)

The degradation pathway of BMZ was reported in the literature [18]. BMZ was hydrolised in acidic condition by refluxing with 1 N HCL solution for 3 h giving its degradant, due to cleavege of the 4,5-azomethine bond, followed by breakage of the 1,2-amide bond to give the benzophenone derivative; “2-(2-amino-5-bromobenzoyl) pyridine” (ABP) in addition to glycine **Fig 1**. On the other hand, when BMZ was exposed to hydrolysis under alkaline-stress condition, by mixing BMZ with 1N NaOH for 3 h under reflux, or by oxidative degradation of BMZ when added to an aqueous solution of 30% H_2_O_2_ for 3 h, this lead to the same degradation product (ABP) being produced. However, at a lower amount than what was produced by acidic hydrolysis. The degradant was separated as mentioned before and well characterized by IR and MS spectrometry [45].

### HPLC development and optimization

Several parameters that can affect chromatographic separation were evaluated and optimized. these parameters included scanning of different wavelengths, experimenting with various types of the aqueous phase, changes of the mobile phase pH, different types and ratio of organic modifier added and flow rate.

#### Optimal scanning wavelength

The ultraviolet spectra of BMZ and ABP at concentrations of 10 μg mL^-1^ each, was found to show a maximum absorption at a wavelength of 232 nm and 236 nm for BMZ and ABP respectively. Therefore, 230 nm was of choice after comparing the two spectra, to offer the highest sensitivity with minimal detected noise.

#### Type of the aqueous phase

Various aqueous phases (water, 0.1% glacial acetic acid, 0.5% phosphoric acid and 0.01% triethylamine) were evaluated in combination with an organic modifier. Water adjusted with phosphoric acid and/or glacial acetic acid was found to show no change on the retention time or peak symmetry of both BMZ and ABP. Also, the addition of 0.01% triethylamine to water gave poor baseline. Thereby, water was selected, having the added advantage of the low cost, having minimal effect on the column, and at the same time gave a better result.

#### Type and ratio of organic modifier

**Fig 2:**
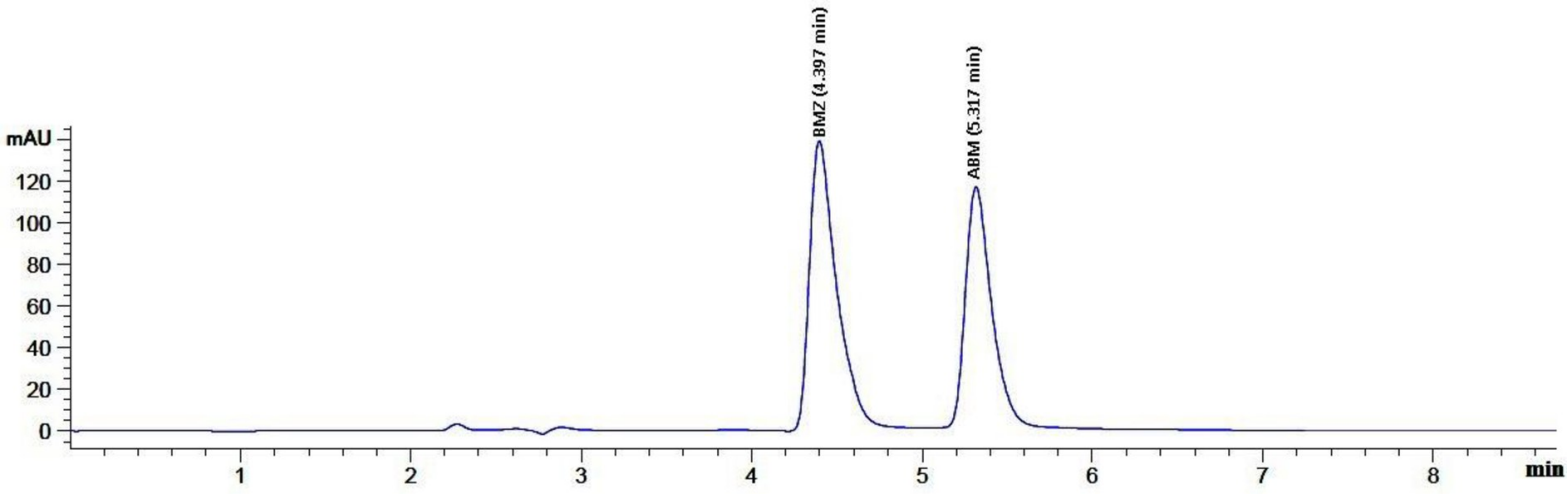
HPLC chromatogram of 12 μg mL-1 of BMZ and 12 μg mL-1 of ABP (Rt = 4.397, 5.317 min respectively). Mobile phase: Methanol: Water (70:30 v/v). Detection: DAD at 230 nm.

Various organic modifiers that included acetonitrile and methanol were evaluated in an attempt to increase the performance of the chromatographic conditions. It was found that by using methanol to the mobile phase, the resolution of components and peak symmetry was noticeably improved. The ratio of methanol also was studied (60–75%, v/v). The retention times of both BMZ and ABP were shortened as the ratio of methanol was increased. We found that concentrations of methanol of 70% (v/v) resulted in the optimal separation of BMZ and ABP with the highest validation parameters, **Fig 2**. However, a further increase in the amount of methanol above 75% (v/v) resulted in poor resolution and overlapped BMZ and ABP peaks.

#### Flow rate

To achieve the best resolution with the shortest separation time, different flow rates of the mobile phase had been evaluated which included flow rates of 1, 1.2 and 1.5 mL min^-1^. A flow rate of 1 mL.min^-1^ was found to be the most optimal.

### HPLC results

The developed isocratic HPLC method for analysis of BMZ and ABP showed to be sensitive, accurate and highly selective. A mobile phase composed of methanol: water (70:30 v/v) was used, with retention times of 4.397, and 5.317 min for BMZ and ABP respectively, **Fig 2**. The total run time required for the analyses of all the compounds of interest was determined to be approximately 6 min. The required short analysis time may have the added advantage of providing a more rapid method for routine analysis of the main investigated drug, with a great level of accuratness and selectivity when compared to previously reported methods [18,19]. Calibration curves for BMZ and ABP were created by drawing the relative peak area against their correspondant concentration. The regression equations were then calculated as follow:

Y=0.1299X+0.0497,r=0.9999forBMZ,

Y=0.1115X+0.0967,r=0.9999forABP,

Where Y is known as relative peak area, while X is the concentration in units of μg mL^-1^, and r corresponds to the correlation coefficient**, Table 1**. This proposed method was further applied for the assay of BMZ in Lexotanil^®^ and Calmepam^®^ tablets (labeled to contain 3 mg of BMZ per tablet). For confirmation of the method validity, standard addition technique was applied, **Table 2.** The results obtained show no interference of additives (included in the dosage forms) with the investigated mixture.

**Table 1:** Validation parameters assay results of the proposed RP-HPLC method for the determination of BMZ and ABP.

Parameter	BMZ	ABP
**Calibration range (μg mL**^**-1**^**)**	1–16	1–16
**Linearity**		
**Slope**	0.1299	0.1115
**Intercept**	-0.0497	0.0967
**Correlation coefficient (r)**	0.9999	0.9999
**Residual standard error**	0.0091	0.0067
**Accuracy (mean ± SD)**	99.96 ± 1.17	99.73 ± 1.13
**Precision (%RSD):**		
**Repeatability** *	1.45	1.62
**Intermediate Precision** *	1.66	2.03
**LOD (μg mL**^**-1**^**)** **	0.20	0.24
**LOQ (μg mL**^**-1**^**)** **	0.60	0.72

* The intra-day and inter-day relative standard deviation of the average of concentrations (2, 6, 10 μg mL^-1^) of both BMZ and ABP.

**Limit of detection and quantitation were computed by calculations (LOD = 3.3×SD/slope, LOQ = 10×SD/slope) [45].

**Table 2:** Determination of BMZ in pharmaceutical formulations by the proposed RP-HPLC method and application of the standard addition technique.

Pharmaceutical formulations	Taken (μg mL^-1^)	Found %* ± SD	Pure added (μg mL^-1^)	Pure found** (μg mL^-1^)	Recovery%**
**Lexotanil**^**®**^ **tablets claimed to contain 3 mg of BMZ (B.N. M1139B01)**	4.00	99.29 ± 1.20	2.00	2.03	101.50
			4.00	4.11	102.75
			6.00	5.95	99.17
	**Mean ± SD**				**101.14 ± 1.82**
**Calmepam**^**®**^ **tablets claimed to contain 3 mg of BMZ (B.N. A506716)**	4.00	98.56 ± 1.56	2.00	1.96	98.00
			4.00	4.09	102.25
			6.00	6.07	101.17
	**Mean ± SD**				**100.47 ± 2.21**

* Average of six determinations.

** Average of three determinations.

### Method validation

The method’s validation was achieved following the guidelines of the ICH [47].

#### Linearity

The method’s linearity was evaluated after the optimized chromatographic conditions were achieved. This was done by collecting the area of the integrated peak of each compound at various concentrations followed by drawing the calibration graphs utilizing the relative peak area of each compound against the compounds corresponding concentrations from which regression equations were constructed. The linearity of the calibration graphs was confirmed by the high value of the correlation coefficient and the low value of the intercept. Additionally, the small values of the residual standard error confirm the fitness and linearity of the proposed method. **Table 1** shows the linearity and range parameters.

#### Accuracy

The percentage recoveries of pure blind samples of the studied compound were used to compute the accuracy of analytical method. In **Table 3,** corresponding regression equations were used to calculate relevant concentrations of compounds. Mean recoveries were almost 100% with standard deviation of less than 1.2% in all cases.

**Table 3:** Results of accuracy for the determination of BMZ and ABP by the proposed RP-HPLC method.

BMZ			ABP		
Taken (μg mL^-1^)	Found* (μg mL^-1^)	Recovery %	Taken (μg mL^-1^)	Found* (μg mL^-1^)	Recovery %
1.00	1.01	101.00	1.00	0.98	98.00
4.00	3.93	98.25	3.00	2.99	99.67
6.00	5.92	98.67	5.00	5.01	100.20
8.00	8.08	101.00	8.00	8.10	101.25
10.00	10.10	101.00	12.00	11.88	99.00
12.00	12.04	100.33	16.00	16.04	100.25
16.00	15.91	99.44	−	−	−
**Mean ± SD**		**99.96 ± 1.17**	**Mean ± SD**		**99.73 ± 1.13**

* Average of three determinations.

A standard addition technique was also performed to both Lexotanil^®^ and Calmepam^®^ tablets, by addition of certain amounts of authentic BMZ, to ensure accuracy. Here we obtained good recoveries (about 99%) and small standard deviation values (less than 2.3% in all cases) suggesting good accuracy and also revealing minimal interference from any excipients within the dosage form; **Table 2**.

#### Precision

Repeatability of the results for three different concentrations (2, 6 and 10 μg mL^-1^) of both BMZ and ABP were performed through replicate analysis (n = 3), within the same day, to estimate intra-day variation. In order to estimate any inter-day variation, triplicate injections were performed on three consecutive days. **Table 1** shows the calculated coefficient of variation (< .2.1% in all cases) at the selected concentration levels.

#### Specificity

The specificity of the proposed method was manifested through a sufficiently good separation of the two compounds, BMZ and ABP at the retention times of 4.397, and 5.317 min, respectively, **Fig 2**.

#### LOD and LOQ

Limits of both detection and quantitation (LOD and LOQ) values for BMZ and ABP by the HPLC method are determined via calculations [48]. The LOD values were equal to 0.2 and 0.24 μg mL^-1^ for BMZ and ABP respectively. The LOQs were also found to be of low values (equal to 0.6 and 0.72 μg mL^-1^ for BMZ and ABP respectively). These low values for LOD and LOQ thereby demonstrating the excellent sensitivity of the suggested chromatographic method; **Table 1**.

#### Robustness

The capability to remain unaffected by minor intentional changes in some parameters of a chromatographic method is frequently used to assess the robustness of the analytical procedure, and indicates its its efficiency during regular use [47]. For the proposed methodology, its robustness was checked, by introducing small changes in the HPLC method. These changes include minor alteration of the flow rate (± 0.2 mL min^-1^), organic strength (± 2%) and performing the HPLC method by different analysts. The values of the relative standard deviation of recovery (%RSD) are found in **Table 4**. The small values of RSD % (< 2%) indicated reliable results concerning the area under the curve.

**Table 4:** Experimental results of robustness testing for the determination of BMZ by the proposed RP-HPLC method.

Parameters	BMZ (%RSD)
**Flow rate (1.2 mL min**^**-1**^**)**	**1.893**
**Flow rate (0.8 mL min**^**-1**^**)**	**1.309**
**Organic strength methanol/water (72: 28%, *v/v*)**	**1.416**
**Organic strength methanol/water (68: 32%, *v/v*)**	**1.940**

#### System suitability

Tests for system suitability were carried out, aiming to check the suitability of the chromatographic system for analysis and its reproducibility. This was conducted by calculating various parameters [49] such as resolution, capacity factor, and peak symmetry, where acceptable results are shown in **Table 5**.

**Table 5:** System suitability parameters of the proposed HPLC method for the determination of BMZ and ABP.

Parameters	BMZ	ABP	Reference value [40]
**Capacity factor (K’)**	1.03	1.45	1–10
**Symmetry factor**	0.95	1.06	~ 1
**Resolution (Rs)**	2.75		R > 2
**Selectivity (α)**	1.21		α > 1
**Number of Theoretical plates (N)**	4229.813	5584.666	Increase with the efficiency of the separation
**HETP Height equivalent to theoretical plate (cm/plate)**	5.9 × 10^−3^	4.5 × 10^−3^	The smaller the value the higher the column efficiency

### Statistical comparison to a reference method

The obtained results by the established HPLC method were compared statistically to the reference HPLC method [15] for Lexotanil® and Calmepam® tablets, where both the Student’s t-test and the variance ratio F-test were used at a 95% confidence level. It was clear from Table 6 that calculated t-test and F-test values are less than that of the tabulated ones. This may indicate that there is no significant difference, regarding both accuracy and precision, between the proposed HPLC method and the reference method.

**Table 6:** Statistical analysis of the current RP-HPLC method and the published HPLC methodology for the determination of BMZ in dosage forms.

Pharmaceutical formulations	Parameters	RP-HPLC	Reported HPLC method **
**Lexotanil**^**®**^ **tablets (B.N. M1139B01)**	**Mean**	99.29	100.04
	**SD**	1.20	1.11
	**Variance**	1.44	1.23
	**N**	6	6
	Student’s *t-*test (2.23)*	1.12	−
	*F-* test (5.05)*	1.17	−
**Calmepam**^**®**^ **tablets (B.N. A506716)**	**Mean**	98.56	100.04
	**SD**	1.56	1.18
	**Variance**	2.43	1.39
	**N**	6	6
	Student’s *t-*test (2.23)*	1.85	−
	*F-* test (5.05)*	1.75	−

* The values between parenthesis are corresponding to the theoretical values of *t* and *F* (p = 0.05).

** HPLC method using C_18_ column, 25% acetonitrile, 45% methanol and 30% ammonium acetate (0.05M) (pH 9) as a mobile phase with UV detection at 240 nm [15].

The proposed stability-indicating RP-HPLC provided the advantage of using a Photo-Diode array detector that is sensitive and accurate due to its ability to provide additional spectral information that is required to identify eluting peaks [50]. The developed HPLC method was depended on isocratic elution and a reversed phase column utilizing an eluent without any electrolyte buffer. As the added buffer solutions to HPLC mobile phase were mostly reported to create several problems, including damage to the pump seals as well as crystal formation in the detector cell and connecting tubing [21].

## Conclusion

The presented isocratic HPLC-DAD method may help in providing high selective, sensitive and reproducible quantitative stability-indicating method, for the analysis of BMZ and ABP simultaneously, at a single wavelength and within a short analysis time. The simple mobile phase composition, compared to previously published methods, with high resolution of both components, adds the advantages of saving time, cost, effort, and protection of the column. Moreover, the method shows low LOD and LOQ values and as a result, can be applied for the detecting low concentrations of BMZ and ABP.

The obtained results indicated that the suggested HPLC method can be included in some of the highly selective and sensitive methods reported for the BMZ and ABP analysis. These merits suggested the usage of the suggested method in analytical quality control (QC) that are routinely performed by regulatory agencies and QC laboratory, without the interference of some of the commonly used dosage form additives.
